# A Pumice Polisher

**Published:** 1871-06

**Authors:** 


### A PUMICE POLISHER.

Dr. R. B. Bole, of Urbana, Ohio, prepares polishers by cutting from blocks of pumice, rods or sticks of the desired size, cut them into sections of one half inch to an inch in length, then drill a small hole in one end, and fasten in it a wooden pin with shellac; this affords an attachment for the port polisher. Now the section can be filed or ground to any desired shape and size, a variety of shapes and sizes will be found useful for dressing fillings and polishing teeth.

